# Crimean-Congo Hemorrhagic Fever Virus Antibodies among Livestock on Corsica, France, 2014–2016

**DOI:** 10.3201/eid2605.191465

**Published:** 2020-05

**Authors:** Sébastien Grech-Angelini, Renaud Lancelot, Olivier Ferraris, Christophe Nicolas Peyrefitte, Nathalie Vachiery, Aurélie Pédarrieu, Armelle Peyraud, Valérie Rodrigues, Denise Bastron, Geneviève Libeau, Bernard Fernandez, Philippe Holzmuller, Renata Servan de Almeida, Vincent Michaud, Noël Tordo, Loïc Comtet, Raphaëlle Métras, François Casabianca, Laurence Vial

**Affiliations:** Groupement Technique Vétérinaire de Corse, Ghisonaccia, France (S. Grech-Angelini);; Unité Mixte de Recherche de Biologie Moléculaire et d’Immunologie Parasitaires, Agence Nationale de Sécurité Sanitaire de l’Alimentation de l’Environnement et du Travail, Institut National de la Recherche Agronomique, École Nationale Vétérinaire d’Alfort, Maisons-Alfort, France (S. Grech-Angelini);; Institut National de la Recherche Agronomique, UR045 Laboratoire de Recherches sur le Développement de l’Élevage, Corte, France (S. Grech-Angelini, F. Casabianca);; Unité Mixte de Recherche Animal Santé Territoires Risques et Écosystèmes, Centre de Coopération International en Recherche Agronomique pour le Développement, Institut National de la Recherche Agronomique, Université de Montpellier, Montpellier, France (R. Lancelot, N. Vachiery, A. Pédarrieu, A. Peyraud, V. Holzmuller, R.S. de Almeida, V. Michaud, R. Métras, L. Vial);; Institut de Recherche Biomédicale des Armées, Brétigny-sur-Orge, France (O. Ferraris, C.N. Peyrefitte);; Institut Pasteur, Dakar, Senegal (C.N. Peyrefitte);; Unité des Virus Émergents, Université Aix Marseille, IRD190, INSERM1207, IHU Méditerranée Infection, Marseille, France (C.N. Peyrefitte);; World Health Organization Collaborative Centre for Arboviruses and Viral Haemorrhagic Fevers, and World Organisation for Animal Health Reference Laboratory for Crimean-Congo Haemorrhagic; Fever Virus and Rift Valley Virus, Institut Pasteur, Paris, France (N. Tordo);; Institut Pasteur de Guinée, Conakry, Guinea (N. Tordo); ID.Vet, Montpellier (L. Comtet)

**Keywords:** antibodies, Crimean-Congo hemorrhagic fever virus, viruses, tickborne infection, livestock, cattle, sheep, goats, Corsica, France, serology, serologic survey, zoonoses, vector-borne infections

## Abstract

We conducted a serologic survey for Crimean-Congo hemorrhagic fever virus antibodies in livestock (cattle, sheep, and goats; N = 3,890) on Corsica (island of France) during 2014–2016. Overall, 9.1% of animals were seropositive, suggesting this virus circulates on Corsica. However, virus identification is needed to confirm these results.

Crimean-Congo hemorrhagic fever (CCHF), the most widespread tickborne viral infection in humans, is a zoonotic disease caused by an orthonairovirus of the *Nairovirida*e family. Symptoms in humans vary from a nonspecific mild febrile syndrome to severe hemorrhagic disease that sometimes leads to death (*1*,*2*), and a wide range of animals are asymptomatic reservoirs (*1*). Corsica is an island of France located in the northwestern part of the Mediterranean Sea (Figure, panel A). Entomologic surveys have revealed that one of the main vectors of CCHF virus (CCHFV), the *Hyalomma marginatum* tick, is abundant on this island (*1*,*3*,*4*). Therefore, we performed a serologic cross-sectional survey to assess the prevalence of antibodies against CCHFV in domestic ruminants on Corsica. This work was approved by the French Ministry of Agriculture (Direction Départementale de la Cohésion Sociale et de la Protection des Populations of Corse-du-Sud and Haute-Corse and General Directorate for Food).

**Figure:**
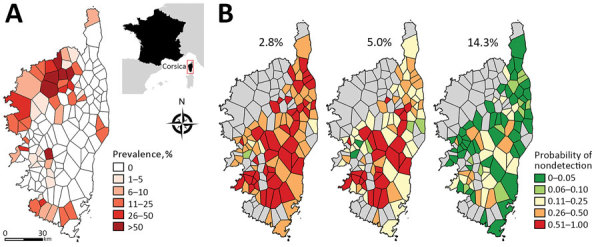
Prevalence and probability of nondetection of antibody against Crimean-Congo hemorrhagic fever virus (CCHFV) in ruminants, Corsica, France, 2014–2016. A) Spatial variability of CCHFV antibody prevalence. Inset indicates location of the island of Corsica in relation to France. B) Probability of nondetection of CCHFV antibody in areas where estimated prevalence was null. Three different probabilities were estimated in accordance with different assumptions of the estimated true seroprevalence, corresponding with the 10% quantile (2.8% seroprevalence), 25% quantile (5.0% seroprevalence), and 50% quantile (14.3% seroprevalence). In this analysis, a Voronoi diagram was used to divide the island into regions; the centroids of Voronoi polygons corresponded to municipalities where blood samples were collected.

As part of national surveillance for animal diseases, veterinarians collected cattle, goat, and sheep blood samples during 2014–2016. In total, 3,890 animals (1,731 cattle, 1,035 goats, 1,124 sheep) were sampled from 269 farms, originating from 46% (137/298) of the municipalities with ruminant farming activities (*3*).

We tested the collected serum samples for the presence of CCHFV IgG using a double-antigen ELISA kit (ID Screen CCHF Double Antigen Multi-species, ID.Vet, https://www.id-vet.com) according to the manufacturer’s instructions (Appendix Figure). For this kit, the 95% CI for sensitivity is 96.8%–99.8%, and 95% CI for specificity is 99.8%–100% (*5*). To confirm ELISA results*,* we sent 35 ELISA-positive and 5 ELISA-negative serum samples to a Biosafety Level 4 laboratory (Laboratory Jean Mérieux, Lyon, France) to be analyzed by the World Health Organization and World Organisation for Animal Health national reference center for CCHFV (Institut Pasteur and Institut de Recherche Biomédicale des Armées, Paris, France). We used the pseudo–plaque reduction neutralization test (PPRNT) (*6*) to measure the neutralizing antibodies against IbAr10200 (same antigen used in ELISA) in triplicate. We included Hazara virus (same serogroup as CCHFV) and Dugbe virus (closely related virus, Nairobi sheep disease serogroup) to detect possible immune cross-reactions. We estimated overall and species-specific IgG prevalence against CCHFV using a β-binomial logistic regression model of data grouped by farm.

The overall estimated seroprevalence was 9.1% (95% CI 6.9%–11.9%); estimated seroprevalence in cattle was 13.3% (95% CI 10.2%–17.3%), goats 3.1% (95% CI 1.4%–7.0%), and sheep 2.5% (95% CI 1.0%–5.9%). CCHFV antibodies were detected across the island; 35.8% (49/137; 95% CI 27.8%–44.4%, estimated by exact binomial test) of the investigated municipalities had >1 positive ELISA test result. Because serum samples were not available from all municipalities, we used Voronoi polygons to draw regional boundaries and estimate the spatial distribution of seroprevalence across the island. Seroprevalence was high in the northwest corner of Corsica; however, most regions lacked evidence of seropositivity (Figure panel A). In areas corresponding to negative polygons, the probability of nondetection of positive serum samples was estimated assuming 3 levels of estimated seroprevalence corresponding with the 10% quantile (2.8% seroprevalence), 25% quantile (5.0% seroprevalence), and 50% quantile (14.3% seroprevalence) (Figure, panel B) and by accounting for sample size. This data shows that if seroprevalence in these regions is <5%, the probability of nondetection is high (Figure, panel B), and if the seroprevalence in these regions is >14.3%, the probability of nondetection is low. Therefore, the chance that we missed hotspots of transmission is highly unlikely.

Of 35 ELISA-positive serum samples tested, none showed neutralizing antibodies against Hazara and Dugbe viruses, and no ELISA-negative serum sample showed neutralizing antibodies against CCHFV, Hazara virus, or Dugbe virus (at lowest dilution 1:20; Appendix Table). Of 35 ELISA-positive serum samples, 23 had neutralizing antibodies against CCHFV at the 1:40 dilution, and 10 remained positive at the 1:80 dilution (including 2 positive at the 1:320 and 1:640 dilutions).

Our serologic survey results suggest CCHFV circulates in livestock on Corsica. Relative discrepancies between ELISA (35 positives) and PPRNT (23 positives) findings might result from their different target epitopes; the ELISA measures total immunoglobulin (neutralizing and nonneutralizing antibodies) and PPRNT just a subset (functional neutralizing antibodies) (*7*). Seroprevalence estimates were higher in cattle than smaller ruminants, probably reflecting that cattle in Corsica are more infested by *Hy*. *marginatum* ticks (*3*).

As of February 2020, CCHFV has not been detected in ticks on Corsica (*8*), and no clinical human case has been reported. The presence of a genetically close and less virulent strain in ticks on Corsica might help explain the lack of these findings. CCHFV was detected in ticks in Spain, where the first human cases were reported in 2016 (*9*), and in a tick collected on a migratory bird in Italy (*10*). Entomologic and epidemiologic investigations to identify the incriminated strain and characterize its spatial distribution are ongoing. This work will be essential to assess the risk for human CCHFV exposure and raise public health awareness on Corsica and in neighboring areas.

Appendix More information on Crimean-Congo hemorrhagic fever virus antibodies among livestock on Corsica, France, 2014–2016.
