# Supplementary material for: Crimean-Congo Hemorrhagic Fever Virus Antibodies among Livestock on Corsica, France, 2014–2016
Source: Emerg Infect Dis. 2020 May;26(5):1041–4. doi: 10.3201/eid2605.191465 (PMC7181912; doi:10.3201/eid2605.191465)
Supplement: Appendix — More information on Crimean-Congo hemorrhagic fever virus antibodies among livestock on Corsica, France, 2014–2016. [file 19-1465-Techapp-s1.pdf]

Article DOI: https://doi.org/10.3201/eid2605.191465

Crimean-Congo Hemorrhagic Fever Virus Antibodies among Livestock on Corsica, France, 2014–2016

Appendix

Appendix Table. Results of PPRNT on 35 ELISA-positive and 5 ELISA-negative sera, using CCHF (in triplicate), Hazara, and Dugbe viruses*

		ELISA test		PPRNT					
				CCHFv					
Sample code	Municipality	PP ELISA	Status ELISA	1st replicate†	2nd replicate	3rd replicate	Consensual titre	Hazara virus	Dugbe virus
1	Vico	292	Positive	Positive	40	80	80	<20	20
2	Vico	263	Positive	Negative	40	80	40	<20	20
3	Vico	175	Positive	Negative	20	20	20	<20	<20
4	Vico	251	Positive	Negative	40	40	40	<20	<20
5	Palasca	147	Positive	Positive	40/80	40	40	20	<20
6	Palasca	177	Positive	Positive	40/80	80	80	20	20
7	Palasca	129	Positive	Positive	80	40	80	20	<20
8	Tolla	221	Positive	Negative	40	40	40	<20	<20
9	Tolla	220	Positive	Negative	20/40	40	40	20	<20
10	Tolla	271	Positive	Negative	20/40	20	20	<20	<20
11	Castifao	194	Positive	Positive	40	40	40	20	<20
12	Castifao	161	Positive	Negative	20	20	20	20	<20
13	Castifao	162	Positive	Negative	20	40	20	<20	<20
14	Olmi Cappella	168	Positive	Positive	80	80	80	20	<20
15	Olmi Cappella	169	Positive	Positive	80	80	80	20	<20
16	Olmi Cappella	213	Positive	Positive	640	320	640	20	<20
17	Pietralba	157	Positive	Negative	80	80	80	20	<20
18	Pietralba	175	Positive	Positive	320	320	320	20	<20
19	Pietralba	157	Positive	Negative	40	40	40	<20	<20
20	Omessa	221	Positive	Positive	40	40	40	<20	20
21	Omessa	190	Positive	Negative	20/40	20	20	<20	<20
22	Omessa	202	Positive	Positive	80	80	80	<20	<20
23	Occhiatana	157	Positive	Negative	40	40	40	20	<20
24	Occhiatana	174	Positive	Negative	80/160	20	20	20	<20
25	Occhiatana	177	Positive	Negative	40	20	20	<20	<20
26	Cargèse	294	Positive	Positive	40	20	40	20	<20
27	Cargèse	235	Positive	Negative	40	20	20	<20	<20
28	Cargèse	317	Positive	Positive	80	<20	80	20	20
29	Cargèse	288	Positive	Negative	80	<20	20	20	20
30	Asco	188	Positive	Negative	20/40	<20	20	<20	20
31	Asco	190	Positive	Negative	40	40	40	<20	20
32	Asco	159	Positive	Negative	20	40	20	<20	<20
33	Casanova	151	Positive	Negative	20/40	40	40	20	<20
34	Casanova	139	Positive	Negative	20/40	80	40	20	<20
35	Casanova	164	Positive	Negative	20/40	20	20	20	<20
36	Vico	3	Negative	Negative	20	40	20	<20	<20
37	Vico	4	Negative	Negative	<20	20	<20	<20	<20
38	Tolla	29	Negative	Negative	20	<20	<20	<20	<20
39	Casanova	3	Negative	Negative	20	<20	<20	<20	<20
40	Olmi Cappella	3	Negative	Negative	<20	<20	<20	<20	<20

*For the first assay with CCHFv, the positive control could not be validated, which avoided the titration of neutralizing antibodies and resulted in a simple positive/negative note. A consensual titre was determined as a result from triplicates. CCHF, Crimean-Congo hemorrhagic fever; CCHFv, Crimean-Congo hemorrhagic fever virus; PPRNT, Pseudo-Plaque Reduction Neutralization Test.
†Positive control not validated.

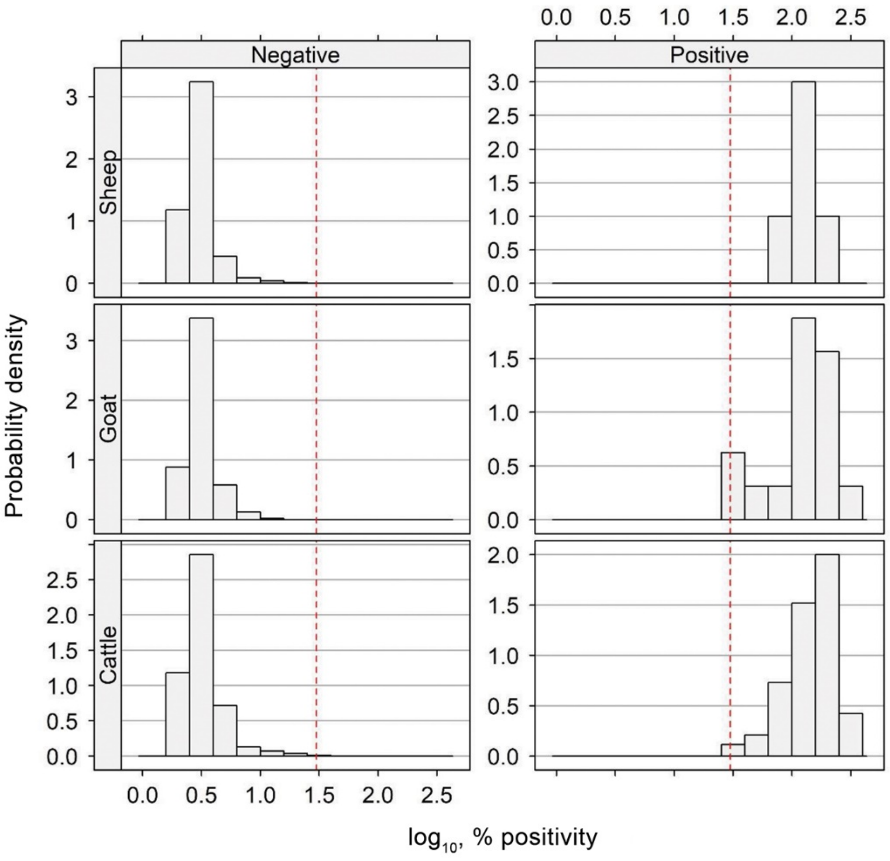

Appendix Figure. ELISA results for the 3,890 serums, stratified by species (sheep, goat and cattle). Results are presented in Percent Probability (PP), which is the optical density obtained for the tested sample divided by the optical density of the positive control of the test, multiplied by 100. Log10 (PP) is represented on the plots. According to the manufacturer's instructions of the test, samples presenting a PP lesser than or equal to 30% (<=1.5 in Log10) are considered negative and those greater than 30% (>1.5 in log10) are considered positive.
